# Dependence on visual information in patients with ACL injury for multi-joint coordination during single-leg squats: a case control study

**DOI:** 10.1186/s13102-024-00875-9

**Published:** 2024-04-17

**Authors:** Minoru Toriyama, Atsuo Nakamae, Takumi Abe, Kazuhiko Hirata, Nobuo Adachi

**Affiliations:** 1 Department of Orthopaedic Surgery, Graduate School of Biomedical and Health Sciences, Hiroshima University, 1-2-3 Kasumi, Minami-ku, 734-8551 Hiroshima, Hiroshima Japan; 2 Department of Rehabilitation, Faculty of Health Sciences, Nihon Fukushi University, Handa, Aichi Japan; 3 Department of Sports Medical Center, Hiroshima University Hospital, Hiroshima, Hiroshima Japan; 4 Department of Rehabilitation, Hiroshima Hiramatsu Hospital, Hiroshima, Hiroshima Japan; 5 Department of Rehabilitation, Division of Clinical Practice and Support, Hiroshima University Hospital, Hiroshima, Hiroshima Japan

**Keywords:** Anterior Cruciate Ligament(ACL), Movement variability, Sample entropy, Joint motion coordination, Single-leg squat, Visual information

## Abstract

**Background:**

The influence of vision on multi-joint control during dynamic tasks in anterior cruciate ligament (ACL) deficient patients is unknown. Thus, the purpose of this study was to establish a new method for quantifying neuromuscular control by focusing on the variability of multi-joint movement under conditions with different visual information and to determine the cutoff for potential biomarkers of injury risk in ACL deficient individuals.

**Methods:**

Twenty-three ACL deficient patients and 23 healthy subjects participated in this study. They performed single-leg squats under two different conditions: open eyes (OE) and closed eyes (CE). Multi-joint coordination was calculated with the coupling angle of hip flexion, hip abduction and knee flexion. Non-linear analyses were performed on the coupling angle. Dependence on vision was compared between groups by calculating the CE/OE index for each variable. Cutoff values were calculated using ROC curves with ACL injury as the dependent variable and significant variables as independent variables.

**Results:**

The sample entropy of the coupling angle was increased in all groups under the CE condition (*P* < 0.001). The CE/OE index of coupling angle variability during the descending phase was higher in ACL deficient limbs than in the limbs of healthy participants (*P* = 0.036). The CE/OE index of sample entropy was higher in the uninjured limbs of ACL deficient patients than in the limbs of healthy participants (*P* = 0.027). The cutoff value of the CE/OE index of sample entropy was calculated to be 1.477 (Sensitivity 0.957, specificity 0.478).

**Conclusion:**

ACL deficient patients depended on vision to control multiple joint movements not only on the ACL deficient side but also on the uninjured side during single leg squat task. These findings underscore the importance of considering visual dependence in the assessment and rehabilitation of neuromuscular control in ACL deficient individuals.

**Supplementary Information:**

The online version contains supplementary material available at 10.1186/s13102-024-00875-9.

## Background

Anterior cruciate ligament (ACL) injury is the one of the most common injury of the knee in sports [1]. The incidence rate of ACL injuries remained relatively stable between 1990 and 2010, especially in females [2]. Moreover, patients who have undergone ACL reconstruction often require revision or suffer an ACL injury on the contralateral side. In the first 5 years after ACL reconstruction, the rate of new ACL injury is higher than the rate of primary ACL injury in the general population [3]. These high ACL injury rates may be due in part to the lack of effective prevention programs before injury and after ACL reconstruction.

Noncontact mechanisms account for 70–76% of all ACL injuries [4] and occur most commonly during dynamic activities involving rapid deceleration and landing [5, 6]. Performing these movements with less risk of ACL injury requires more skillful neuromuscular control. In previous studies, neuromuscular control systems have often been represented by the movement of the centre of pressure (COP). Fernandes TL et al. observed that during single-leg standing and squat tasks, athletes with ACL injuries exhibited greater lateral shifts in the COP than did healthy subjects [7]. Similarly, Nematollahi M et al. reported that the trembling component of the COP, which reflects peripheral systems such as muscle activity, was significantly greater in individuals with ACL deficiency under both single-leg and double-leg conditions, indicating increased instability [8]. Bodkin SG et al. reported no significant difference in the average velocity of the COP during one-legged stance postural control between patients who had undergone ACL reconstruction and healthy subjects, suggesting that ACL reconstruction may restore some aspects of postural control to preinjury levels [9]. However, Steffen K et al. found no correlation between COP movement velocities, both anterior-posterior and lateral, during static and dynamic postural control and the risk of ACL injury in female elite handball and soccer players [10]. This indicates that poor movement specific to those at high risk of ACL injury is inadequately measured by COP and that it is difficult to identify features of the neuromuscular control system. Moreover, several studies suggest that noncontact ACL injury results from multi-plane joint moment caused by multi-directional ground reaction forces [5, 11, 12]. These studies show that controlling joint motion across multiple joints could lead to postural stabilization and prevention of ACL injury. The nonlinear analyses of motion variability associated with ACL injury or reconstruction have focused on two different joints or two joint motions [13–15]. On the other hand, it has been shown that more than three joint motions, including others in the knee joint, may be involved in the risk of noncontact ACL injury. Video analysis at the time of ACL injury reported low hip flexion angles [16], and weakness of the hip abductor muscle strength was a risk factor for non-contact ACL injuries [17]. In addition, a decrease in absorption in the lower extremity due to a smaller hip flexion angle motion [18], this is an energy absorption strategy that relies on distal joints such as the ankle joint and may increase the knee valgus motion [19]. It is speculated that the combined occurrence of these factors increases the risk of ACL injury. These joint movements can be controlled by muscles, unlike knee valgus motion which does not have a primary active muscle, so skillful control of these joint movements may be useful in preventing ACL injury.

Recent studies have shown that coordination patterns change depending on the availability of visual information [20]. Studies of stability control related to ACL injury have examined the influence of vision in static assessments such as quiet standing or one-legged standing. In several previous reports, COP deviation in patients after ACL reconstruction was greater with closed eyes than with open eyes, and these values showed a greater range of elevation than did those on the healthy or uninjured side [21–23].. However, prevention of ACL injuries or revision after ACL reconstruction requires stable postural control in more dynamic situations. Trulsson et al. reported deviations in muscular activity between the injured and noninjured sides in individuals with ACL injuries during single-leg squats, suggesting altered sensorimotor control [24]. Therefore, the influence of vision on the neuromuscular control system in more complex tasks, such as the single-leg squat, should be considered.

The purpose of this study was to reveal differences in neuromuscular control in ACL-deficient patients during single-leg squats with different visual information via nonlinear analysis for multiple joint movements. We hypothesized that ACL-deficient patients would exhibit more variability during movement in both of injured side and uninjured side, and that reduced visual information would further manifest their characteristic movements variability.

## Methods

### Participants

This study was approved by the Ethical Review Committee for Medical Research Involving Human Subjects in accordance with the Declaration of Hiroshima University (ID number: C-274-1). Twenty-three patients with non-contact ACL injury (23 affected knees) aged from 16 to 42 years (11 males and 12 females; mean age, 21.7 ± 6.9 years old) participated in this study. The recruitment period for this study was between July 2019 and June 2022. The inclusion criteria were as follows: outpatients of the Department of Orthopaedic Surgery, Hiroshima University Hospital, who completed junior high school or other courses, diagnosed by an experienced orthopaedic surgeon as having ACL injury based on MRI imaging findings and physical findings, requiring ACL reconstruction, and able to walk alone. The surgeon was trained to uniformly evaluate the physical examination findings, including the pivot shift test and the Lachman test. Patients were excluded if they had any of the following: under 16years old, bilateral ACL injury, history of lower limb injuries within 2 years, ligament reconstruction within 2 years, knee or hip joint arthroplasty or high tibial osteotomy, neuromuscular disorder, history of stroke or cardiovascular disease, or any other gait abnormalities. For comparison, 23 healthy subjects matching age and body size with no history of neuromuscular disorder or orthopaedic problems in the lower limbs participated. Participant characteristics are shown in Table 1. All participants in this study gave informed consent using documentation and signed a consent form.

**Table 1:** Characteristics of participants

	Healthy volunteer (*n* = 23)	ACL injured patients (*n* = 23)	P value
Age (years old)	21.7 ± 5.5	21.7 ± 6.9	n.s.
Sex(female/male)	11/12	11/12	n.s.
Body height(cm)	166.7 ± 9.2	166.4 ± 8.5	n.s.
Body weight(kg)	63.3 ± 14.8	67.3 ± 10.4	n.s.
BMI(kg/m^2^)	22.6 ± 3.9	24.3 ± 3.2	n.s.
Time since injury (months)	-	24.3 ± 19.4	-
IKDC score	99.3 ± 1.9	68.2 ± 13.0	< 0.001*
Tegner Activity Score	5.96 ± 1.36	4.39 ± 1.84	< 0.001*
Lysholm Knee Scoring Scale	99.8 ± 1.0	85.0 ± 10.0	< 0.001*
KOOS			
Symptoms	100 ± 0	85.6 ± 9.6	< 0.001*
Pain	99.4 ± 2.9	87.2 ± 8.0	< 0.001*
Function, daily living	100 ± 0	93.6 ± 6.0	< 0.001*
Function, sports and recreational activities	99.8 ± 1.0	62.0 ± 20.3	< 0.001*
Quality of Life	100 ± 0	63.1 ± 16.4	< 0.001*

mean ± S.D., *: *p* < 0.05, unpaired t test, n.s.: not significant

ACLD: anterior cruciate ligament deficient

IKDC: International Knee Documentation Committee

KOOS: Knee injury and Osteoarthritis Outcome Score

A prior power analysis for sample size was performed with G*Power (version 3.1; Franz Faul, Kiel University, Kiel, Germany); for an effect size of 0.3, power of 0.80, an α level of 0.05, and numerator degrees freedom of 1 and 2, number of groups of 6; a total of 90 and 111 samples for main effects, and 111 samples for interactions were needed, respectively. Therefore, there was a minimum of 23 samples for each condition and for each group considering possible dropout in this study.

### Procedure

Kinematic data on the patient motion were acquired using a three-dimensional motion analysis system (VICON NEXUS; Vicon Motion Systems, Oxford, UK) with 16 infrared cameras (Vicon Motion Systems, Oxford, UK) operating at 200 Hz. Before each measurement session, devices were calibrated, and the mean calibration residuals for trials were under 1.00 mm.

Infrared-reflecting markers 14 mm in diameter were attached to 45 landmarks including the left front head, right front head, left back head, right back head, 7th cervical vertebrae, 10th thoracic vertebrae, clavicle, sternum, right back, bilaterally acromion, lateral epicondyle approximating the elbow joint, wrist bar thumb side, and pinkie side, head of the 2nd metacarpus, anterior superior iliac spine, posterior superior iliac spine, great trochanter, lateral aspects of the thighs, lateral and medial epicondyles of the femur, lateral aspects of the shanks, lateral and medial condyles of the tibia, lateral and medial malleoli, head of the 2nd metatarsal heads, and the calcaneal tuberosity. Motion trials were captured as the participant performed single-leg squats (SLSs). Participants performed the actual task after completing a minimal set of fewer than five consecutive SLSs with their eyes open as a preliminary exercise. Participants were instructed to perform 12 SLSs with their hands on hips; requirements for the flexion angles of the joints and the depth of the squat were not specified. The SLSs were conducted in sync with a metronome set at 120 bpm, such that the metronome emitted a clicking sound once at the lowest position of the squat and once at the highest position. Participants performed the exercises under two randomized order conditions; the eyes-opened (OE) and eyes-closed (CE), with the supporting leg, which was defined as the lower limb on the supporting side when kicking a ball, in healthy subjects (Healthy) and both the ACL-deficient (ACLD) side and contralateral uninjured side (Uninjured) in patients with ACL injury. Successful trials were those in which the participants performed 12 repetitions without the opposite lower limb touching the ground and performed in rhythm. The first and last one each were excluded, and the 10 SLSs were analysed.

### Data processing

The lower limb joint angles and centre of mass (COM) were calculated using the processing software Body-Builder (Vicon Motion Systems, Oxford, UK) based on collected marker coordinates. The centre of a participant’s ankle joint was estimated as the midpoint between the malleoli, while the knee joint centre was estimated as the midpoint between the lateral and medial epicondyles of the femur and the lateral and medial condyles of the tibia. The hip joint centre was estimated based on a previous study [25]. The collected marker coordinates were used to define the respective local coordinate systems of the fifteen-point body link model consisting of the head, thorax, both upper arms, both lower arms, hands, pelvis, both thighs, both shanks, and both feet. The position of the centre of mass position for each segment was calculated based on body inertia characteristics in a report by Okada et al. [26], and all composite centres of mass for all segments were used as the whole-body COM. A single squat was identified as the combined descending and ascending phases of a SLS indicated by the COM vertical displacement between the vertical maximum position.

### Multiple joint coordination

Hip flexion-extension, hip abduction-adduction, and knee flexion-extension motions are associated with ACL injury [6]. The coordination of these three joint motions, hip flexion (+)–extension (-), hip abduction (+)–adduction (-) and knee flexion (+)–extension (-), and the coupling angle (CA) were obtained from the Appendix.

The COM data were divided into ascending and descending phases, and the coupling angle variability (CAV) was calculated for each phase by the Appendix, and the average of 10 SLSs. The sample entropy (SaEn) of the CA was calculated with embedding dimension, and tolerance was set to 2 and 0.2 × SD, respectively [27]. Non-linear analysis processing was conducted in open-source Python (version 3.9) under Jupyter Notebook with pandas, nolds, numpy, sklearn and Anaconda libraries.

### Dependence of visual information

To examine the effect of visual acuity, the CE/OE index was calculated for each variable by dividing the CE value by the OE value. Values close to 1 suggest a minimal influence of visual information on balance, while values greater than 1 indicate a greater dependency on vision.

### Statistical analysis

The statistical analyses were performed using IBM SPSS 25.0 (SPSS Inc., Chicago, IL, USA). Differences in physical characteristics between groups in the participants were tested with an unpaired t-test. A two-way factorial analysis of variance (ANOVA) was performed to assess the effects of group (Healthy vs. ACLD vs. Uninjured) and condition (eyes-open and eyes-closed) on COP values, CAV and SaEn. One-way ANOVA was performed for the CE/OE index for each variable. All variables are presented as the mean and SD. If significant main effects or interactions were identified using ANOVA, post hoc pairwise comparisons using the Tukey‒Kramer multiple comparisons test were then performed.

Finally, the capacities of dependence on visual information indicators for predicting ACL-injured risk were compared via area under the receiver-operating characteristics (ROC) curves (AUC) analysis. We analysed variables significantly different from healthy subjects for the uninjured side limb of ACL-injured individuals, rather than the injured leg, in order to identify the potential risk of ACL injury. The cutoff value was defined as the point at which the Youden Index of the ROC curve was the largest. All p values were two–sided and *p* < 0.05 was considered statistically significant.

## Results

### Effects of ACL injury and condition on CAV and SaEn

Significant condition-specific effects were observed for ascending-phase CAV (*p* < 0.001, F = 33.86), descending-phase CAV (*p* = 0.048, F = 3.99) and SaEn CA (*p* < 0.001, F = 52.02). Conversely, the main effect of group failed to reach statistical significance. All results of two-way ANOVA are shown in Table 2.

**Table 2:** Effects of group and condition for CAV and SaEn

	Healthy		ACLD		Uninjured		F value			P value		
	OE	CE	OE	CE	OE	CE	Group	Condition	Group*Condition	Group	Condition	Group*Condition
Ascending phase CAV	0.05±0.03	0.1±0.07	0.04±0.04	0.1±0.06	0.04±0.05	0.1±0.07	0.16	33.86	0.005	0.85	**< 0.001** ^*^	0.99
Descending phase CAV	0.13±0.06	0.14±0.06	0.11±0.05	0.14±0.05	0.13±0.06	0.15±0.06	1.06	3.99	0.60	0.349	**0.048** ^*^	0.55
SaEn CA	0.03±0.01	0.04±0.01	0.03±0.01	0.04±0.01	0.03±0.01	0.04±0.01	0.27	52.02	1.67	0.77	**< 0.001** ^*^	0.19

mean ± S.D.

*: significant effect in A two-way factorial analysis of variance

ACLD: anterior cruciate ligament deficient, OE: open eyes, CE: closed eyes, CAV: coupling angle variability, SaEn: sample entropy, CA: coupling angle

### Effects of visual information for CAV and SaEn

**Fig. 1:**
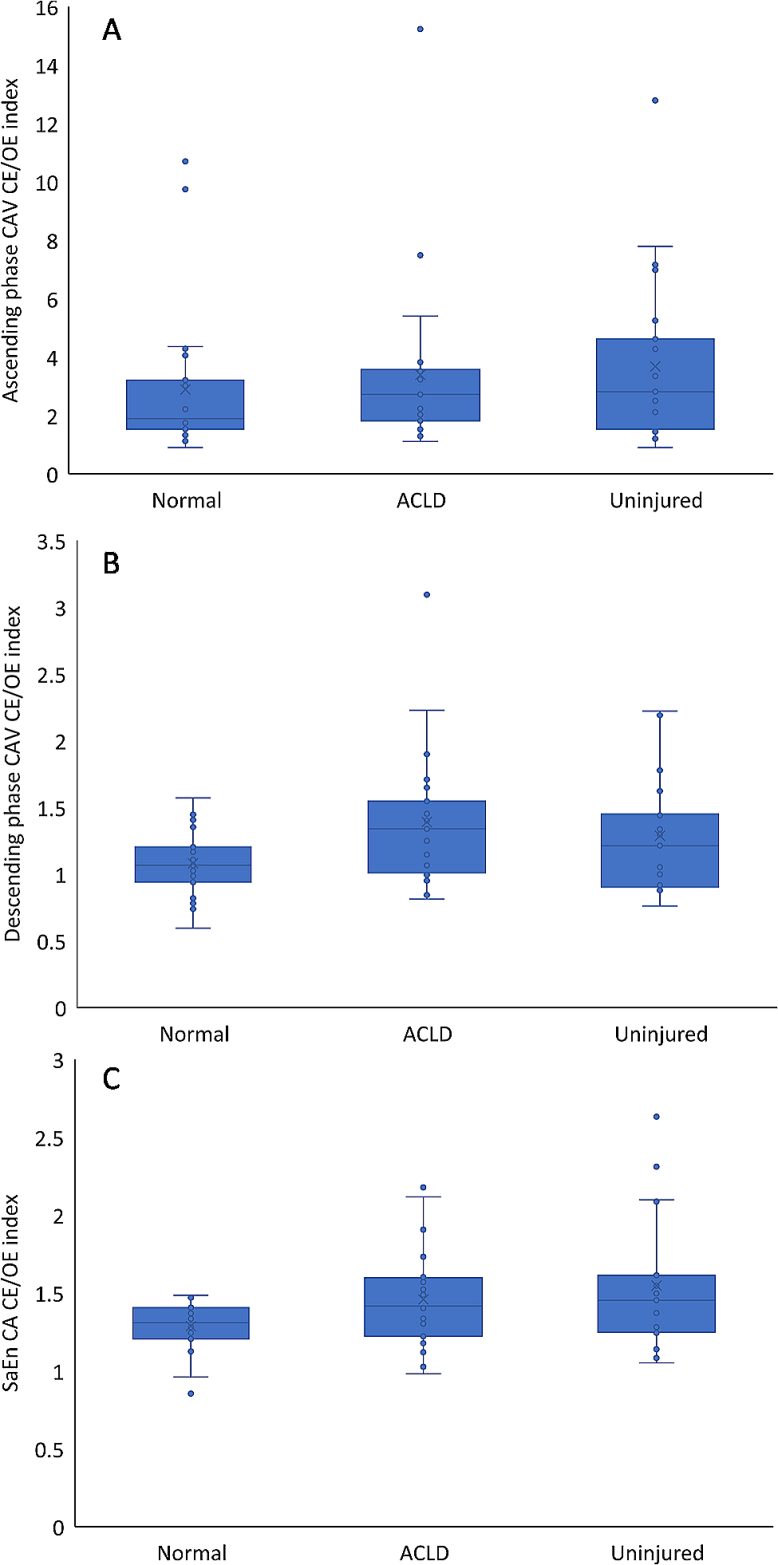
CE/OE index of CAV and SaEn. Group mean ± 95% confidence intervals and individual participant mean data. Data presented and abbreviations are as follows: Ascending phase CAV CE/OE index as (**A**), Descending phase CAV CE/OE index as (**B**), SaEn CA CE/OE index as(**C**)

Group mean ± 95% confidence intervals, along with individual participant mean outcome measures are presented in Fig. 1, with full statistical analysis reported in Table 3. The CE/OE index of CAV during the descending phase in the ACLD was higher than that in healthy participants (95% CI 0.017-0.60; *P* = 0.036; Table 3). The CE/OE index of SaEn on the uninjured side was higher than that of healthy participants (95% CI 0.031–0.49; *P* = 0.027; Table 3.)

**Table 3:** Effects of visual information for CAV and SaEn

CE/OE index	Healthy	ACLD	Uninjured	F value	P value
Ascending phase CAV	2.89±2.47	3.39±2.89	3.68±2.72	0.49	0.617
Descending phase CAV	1.08±0.25	**1.39±0.49** ^**†**^	1.29±0.43	3.34	**0.036** ^*^
SaEn CA	1.29±0.16	1.46±0.31	**1.55±0.43** ^**†**^	3.82	**0.027** ^*^

mean ± S.D.

*: indicate significant in one-way ANOVA

†: indicate significant difference compared with Healthy, post-hoc: Tukey-Kramer multiple comparisons test

ACLD: anterior cruciate ligament deficient, OE: open eyes, CE: closed eyes,

CAV: coupling angle variability, SaEn: sample entropy, CA: coupling angle

### ROC curve analysis

**Fig. 2:**
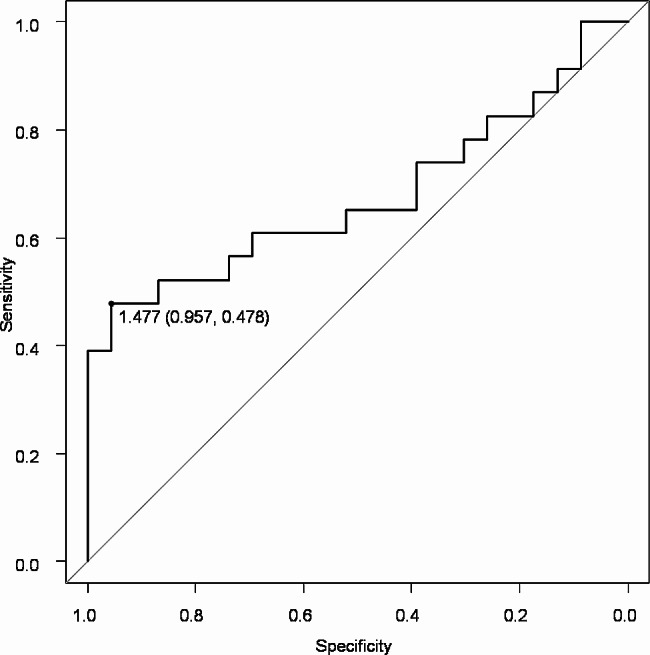
ROC curve of CE/OE index of SaEn. The ROC curves displayed CE/OE index of SaEn cutoff of 1.477 (sensitivity 0.957, specificity 0.478), and AUC was 0.677(95% CI 0.513–0.84)

ROC curve analysis by CE/OE index of SaEn is shown in Fig. 2. The cutoff of the CE/OE index of SaEn was calculated to be 1.477 (sensitivity 0.957, specificity 0.478), and AUC was 0.677(95% CI 0.513–0.84).

## Discussion

The most important finding in this study is that patients with ACL injuries have contralateral neuromuscular control system dysfunction, indicating that stable and continuous joint motion is difficult at multiple joints. These results may quantify the potential risk of ACL injury in individuals with current ACL injuries and may be useful for preventing not only future reinjury of the reconstructed ACL but also new injury of the contralateral ACL.

Our study results showed that, in both healthy subjects and those with ACL injuries, the variability in postural control during SLSs was greater when the subjects’ eyes were closed than when they were open. Specifically, we observed an average increase in variability of 136.8% during the ascending phase and 117.3% during the descending phase, as measured by the CAV, and an average increase of 141.8% in the SaEn CA. This finding indicates greater variability in postural control during dynamic motor tasks with eyes closed. However, there was no difference in variability under both the open-eyes and closed-eyes conditions between the two groups. These results are consistent with the finding of Dingenen et al., who demonstrated no significant difference in single-leg stance COP stability among healthy, ACL-injured, and contralateral limbs under both open-eyes and closed-eyes conditions [28]. In contrast to our findings, prior studies have reported that compared with healthy subjects, individuals with ACL injuries exhibited impaired postural control on not only the injured side but also the contralateral side during static tasks such as static standing and single-leg standing [29, 30]. We speculate that this difference is because we selected the SLS, which is a more dynamic task. Among several dynamic motor tasks, the SLS is more susceptible to dual-task effects [31]. Our study results indicate that it is not appropriate to compare the absolute values of the variability in the movement of SLSs between open and closed eyes. However, several reviews have indicated that a more dynamic assessment is needed for the prevention of noncontact ACL injuries and reinjury [32, 33]. This shows that a novel dynamic postural control assessment index is needed to detect the risk that ACL-injured patients have.

In this study, CE/OE was evaluated to quantify the reliance on visual information in ACL-injured subjects. The results revealed greater variability on the ACLD limb than healthy subjects’ limb during the descending phase of CAV and on the uninjured limbs than healthy subjects’ limb during the entire SaEn. These results suggest that ACL-injured subjects use a postinjury adapted or preinjury potentially visually dependent movement strategy. ACL-injured patients are known to have different motor patterns on the contralateral side compared to healthy subjects [34], and lack of visual information promotes a more rigid movement pattern [35]. This might show that ACL-injured patients exhibit more rigid movement patterns when required to perform the dynamic task of collecting more sensory information due to the loss of visual information. In order to understand the clinical significance of these variables, we used ROC analysis to determine the cutoff risk of ACL injury on the uninjured side limb, which is less susceptible to changes in joint motion due to ACL injury. The cutoff value was 1.477, and the sensitivity was reasonably high, indicating the possibility of screening for ACL injury risk, and although the specificity is low, it is useful for clinically screening individuals for prevention programs. ACL-injured limbs demonstrated lower kinesthesia [36], fewer somatosensory evoked potentials than healthy subjects [37], and a lack of muscle coactivation modulation [38]. ACL-injured individuals might be implementing adaptations to the reduced afferent input at the knee joint due to ACL deficiency that increases afferent joint sensory input information by increasing joint motion variability [39, 40]. In addition to these studies, the lack of visual information for the ACL-injured subjects may also increase variability in other multi-joint movements, including the hip joint that we evaluated, to increase dynamic afferent joint sensory input. Moreover, Diekfuss et al. evaluated altered brain connectivity that may have predisposed athletes to ACL injury and reported that those who went on to experience an ACL injury had decreased functional connectivity between the left primary sensory cortex and right posterior lobe of the cerebellum [41]. These previous reports suggest that ACL-injured patients have different neuromuscular control systems than healthy subjects even before ACL injury, which may have been highlighted by the visual deficits and dynamically unstable motor tasks in the present study. Future research exploring optimal variability in multiple joints will provide a better understanding of ACL injury prevention.

There are three limitations to this study. First, it is not clear when differences in motor control on the uninjured side occur in ACL-injured patients. The subjects with ACL injuries had lower Tegner activity scores than did the healthy subjects, which may indicate that inactivity affects postural control. In addition, it is known that ACL-injured individuals experience a variety of changes due to injury, one of which affects the contralateral lower extremity [42]. To verify this, a prospective cohort study is needed to determine if there are any differences in the contralateral lower extremity in future ACL injury survivors. Second, this study does not fully demonstrate whether ACL deficiency causes differences in movement characteristics on the ACL-injured side. Therefore, it is necessary to clarify the effects of afferent and efferent neuromuscular control system functions, including tests of proprioceptive function and evaluation of muscle activity. To investigate the effects of ACL deficiency, it is necessary to examine whether changes in movement variability occur when knee joint stability is improved through prospective studies after ACL reconstruction. The application of principal component analysis for dimensionality reduction in this study potentially constrained the multidimensional analysis of the data. Consequently, critical insights into the unique joint motion characteristics of individuals with ACL injuries, such as variability in motion during specific time intervals and the interplay between different conditions, might not have been fully captured. To overcome these limitations, future research should include analytical techniques capable of identifying variability across specific periods, such as Statistical Parametric Mapping [43], to provide a more comprehensive understanding of the factors contributing to ACL injury risk.

## Conclusion

Subjects with ACL injuries exhibit increased variability and dependence on visual information during SLSs, as indicated by higher CE/OE indices in multiple joint CAV and SaEn than healthy subjects. These findings underscore the importance of considering visual dependence in the assessment and rehabilitation of neuromuscular control in ACL-deficient individuals.

### Electronic supplementary material

Below is the link to the electronic supplementary material.

Supplementary Material 1

## Data Availability

The data sets used and/or analyzed in this study are available from the corresponding author if reasonably requested.

## Abbreviations

ACL: Anterior cruciate ligament
OE: Open eyes
CE: Closed eyes
COP: Center of pressure
BMI: Body mass index
MRI: Magnetic resonance imaging
SLS: Single-leg squats
ACLD: Anterior cruciate ligament deficient
COM: Centre of mass
CA: Coupling angle
CAV: Coupling angle variability
SaEn: Sample entropy
AUC: Area under the curve
ROC: Receiver-operating characteristic
ANOVA: Analysis of variance
