# Supplementary material for: Dependence on visual information in patients with ACL injury for multi-joint coordination during single-leg squats: a case control study
Source: BMC Sports Sci Med Rehabil. 2024 Apr 17;16:87. doi: 10.1186/s13102-024-00875-9 (PMC11022393; doi:10.1186/s13102-024-00875-9)
Supplement: Supplementary file 1 — Supplementary Material 1 [file 13102_2024_875_MOESM1_ESM.docx]

# **Appendix**

The coordination of multiple joint motions was obtained by the following processing steps: (1) Build a 10 times squats × 3 joint angle matrix for each individual. (2) The principal component analysis (PCA) was computed through a singular value decomposition of the input matrix’s covariance matrix, and dimensionality removal was performed and aggregated into two components. For each subject, two components explained over 90% of the variance. (3) For the established PCs, the coupling angles were calculated as follows. For each instant (*i*) during the 10 squats, the coupling angle (*γi*) was calculated based on the 1st principal component (PC*_1(i)_*, PC*_1(i+1)_*) and the 2^nd^ principal component (PC*_2(i)_*, PC*_2(i+1)_*) according to Formulas (1) and (2):

$\gamma_{i}=Atan\left( \frac{{PC}_{2\left( i+1 \right)}-{PC}_{2\left( i \right)}}{{PC}_{1\left( i+1 \right)}-{PC}_{1\left( i \right)}} \right)\cdot\frac{180}{\pi} ,when {PC}_{1\left( i+1 \right)}-{PC}_{1\left( i \right)}>0$ (1)

$\gamma_{i}=Atan\left( \frac{{PC}_{2\left( i+1 \right)}-{PC}_{2\left( i \right)}}{{PC}_{1\left( i+1 \right)}-{PC}_{1\left( i \right)}} \right)\cdot\frac{180}{\pi}+180 ,when {PC}_{1\left( i+1 \right)}-{PC}_{1\left( i \right)}<0$ (2)

The following conditions (3) were applied:

$\gamma_{i}=\left\{ \begin{aligned} \gamma_{i}=90 {PC}_{1\left( i+1 \right)}-{PC}_{1\left( i \right)}=0 and {PC}_{2\left( i+1 \right)}-{PC}_{2\left( i \right)}>0 \\ \gamma_{i}=-90 {PC}_{1\left( i+1 \right)}-{PC}_{1\left( i \right)}=0 and {PC}_{2\left( i+1 \right)}-{PC}_{2\left( i \right)}<0 \\ \gamma_{i}=-180 {PC}_{1\left( i+1 \right)}-{PC}_{1\left( i \right)}< 0 and {PC}_{2\left( i+1 \right)}-{PC}_{2\left( i \right)}=0 \\ \gamma_{i}=Underfined {PC}_{1\left( i+1 \right)}-{PC}_{1\left( i \right)}=0 and {PC}_{2\left( i+1 \right)}-{PC}_{2\left( i \right)}=0 \end{aligned} \right.$ (3)

The coupling angle (*γi*) was corrected to present a value between 0° and 360° according to (4)

$\gamma_{i}=\left\{ \begin{aligned} \gamma_{i}+360 \gamma_{i}<0 \\ \gamma_{i} \gamma_{i}\geq0 \end{aligned} \right.$ 　　(4)

The coupling angle (CA) was averaged through 10 squats for each participant using circular statistics (Eqs. (5)-(7)).

$\bar{x}=\frac{1}{n}\sum_{i=1}^{n} \cos\gamma_{i}$ (5)

$\bar{y}=\frac{1}{n}\sum_{i=1}^{n} \sin\gamma_{i}$ (6)

where n is the data points across the descent phase and ascension phase in the SLS. To correct for the average CA between presenting a value between 0° and 360°, the following condition (7) was applied.

$\bar{\gamma_{i}}=\left\{ \begin{aligned} Atan\left( \frac{\bar{y}_{i}}{\bar{x_{i}}} \right)\cdot\frac{180}{\pi} \bar{x}_{i} >0 \mathrm{and} \bar{y}_{i}>0 \\ Atan\left( \frac{\bar{y}_{i}}{\bar{x_{i}}} \right)\cdot\frac{180}{\pi}+180 \bar{x}_{i}<0 \\ Atan\left( \frac{\bar{y}_{i}}{\bar{x_{i}}} \right)\cdot\frac{180}{\pi}+360 \bar{x}_{i} >0 \mathrm{and} \bar{y}_{i}<0 \\ 90 \bar{x}_{i}=0 \mathrm{and} \bar{y}_{i}>0 \\ -90 \bar{x}_{i}=0 \mathrm{and} \bar{y}_{i}<0 \\ Underfined \bar{x}_{i}=0 \mathrm{and} \bar{y}_{i}=0 \end{aligned} \right.$ (7)

The length of the average coupling angle $r_{i}$ and coupling angle variability (*CAV*) were calculated using circular statistics (Eqs. (8), (9))

$\bar{r}=\sqrt{\bar{x}^{2}+\bar{y}^{2}}$ (8)

$CAV=\sqrt{2\cdot\left( 1-\bar{r} \right)\cdot}\frac{180}{\pi}$ (9)
